# Percutaneous Coronary Intervention for Left Main Disease in High Bleeding Risk: Outcomes from a Subanalysis of the Delta 2 Registry

**DOI:** 10.3390/jcdd12050179

**Published:** 2025-05-11

**Authors:** Giulia Botti, Francesco Federico, Emanuele Meliga, Joost Daemen, Fabrizio D’Ascenzo, Davide Capodanno, Nicolas Dumonteil, Didier Tchetche, Nicolas M. Van Mieghem, Sunao Nakamura, Philippe Garot, Andrejs Erglis, Ciro Vella, Corrado Tamburino, Marie Claude Morice, Roxana Mehran, Matteo Montorfano, Alaide Chieffo

**Affiliations:** 1 Interventional Cardiology Unit, IRCCS San Raffaele Scientific Institute, 20132 Milan, Italy; 2 School of Medicine, Università Vita-Salute San Raffaele, 20132 Milan, Italy; 3 Interventional Cardiology Unit, Cardinal Massaia Hospital, 14100 Asti, Italy; 4 Department of (Interventional) Cardiology, Thoraxcenter, Erasmus University Medical Center, 3015 GD Rotterdam, The Netherlands; 5 Cardiovascular and Thoracic Department, Division of Cardiology, A. O. U. Città della Salute e della Scienza, 10126 Turin, Italy; 6 Division of Cardiology, Azienda Ospedaliero Universitaria Policlinico “G. Rodolico-San Marco”, University of Catania, 95123 Catania, Italy; 7 Clinique Pasteur, 31076 Toulouse, France; 8 Department of Cardiovascular Medicine, New Tokyo Hospital, Matsudo 270-2232, Japan; 9 Institut Cardiovasculaire Paris Sud (ICPS), Hôpital Jacques Cartier, Ramsay-Santé, 91300 Massy, France; 10 Latvian Centre of Cardiology at Pauls Stradins Clinical University Hospital, 1002 Riga, Latvia; 11 The Zena and Michael A. Wiener Cardiovascular Institute, Icahn School of Medicine at Mount Sinai, New York, NY 10029, USA

**Keywords:** coronary artery disease, risk factors, frailty

## Abstract

High bleeding risk (HBR) is a challenge in patients with complex coronary lesions undergoing percutaneous coronary intervention (PCI). This study investigates HBR in a wide and comprehensive cohort of patients undergoing left main (LM) PCI and reports in-hospital and follow-up outcomes. The analysis was performed on data from the DELTA (Drug Eluting Stent for Left Main Coronary Artery) 2 Registry, which included patients who underwent LM PCI at 19 centres worldwide. The patients were defined to be at HBR if ≥1 major criterion or ≥2 minor criteria from the Academic Research Consortium (ARC) were met. The primary endpoint was a composite of all-cause death, myocardial infarction (MI) or cerebrovascular accident (CVA) at median follow-up. A total of 1531 patients were included, and the rate of HBR was 65.8%. Besides the different clinical characteristics embedded in the ARC definition, HBR patients had higher prevalence of acute coronary syndrome (ACS) at presentation (49.2% vs. 26.8%, *p* < 0.001) and experienced higher in-hospital mortality (1.8% vs. 0.2%; *p* = 0.029) and MI (5.0% vs. 2.1%, *p* = 0.009). The median follow-up was 473 days. The rate of the primary endpoint was more than three times higher in HBR patients (20.8% vs. 6.1%; HR 3.3; 95%CI: 2.2–4.8) and driven by all-cause death at multivariate regression analysis. Conversely, no significant difference in target lesion revascularization and probable or defined stent thrombosis was reported. HBR patients undergoing LM PCI experienced higher rates of all-cause death at follow-up; similar outcomes were also reported in-hospital.

## 1. Introduction

Percutaneous revascularization of left main (LM) disease has garnered significant attention over the last decades as a safe and effective alternative to a coronary artery bypass graft (CABG) in an increasing number and variety of patients [1]. This growing interest is primarily attributed to advancements in stent technology and operator expertise, making it a viable option for an increasing number of patients, facing diverse selection and management challenges [2].

LM percutaneous coronary intervention (PCI) faces inherent high-risk characteristics, strongly associated with its extensive vascular territory, which may potentially encompass up to 80% of the total myocardium. Consequently, adverse events during procedures and the enduring effectiveness of revascularization play a crucial role in shaping patient prognosis, which is currently characterized by lower survival rates compared to patients with lesions in alternative anatomical locations [3].

Although CABG may represent a more durable treatment solution for LM revascularization, it is essential to acknowledge the heightened periprocedural complications associated with this approach. In this perspective, PCI emerges as a compelling alternative, especially for individuals deemed inoperable or facing high surgical risk, such as those of advanced age with significant comorbidities [4,5]. Among these, patients at high bleeding risk (HBR) present a notable challenge in devising optimal revascularization strategies for LM coronary artery disease (CAD). In fact, beyond susceptibility to bleeding and subsequent morbidity and mortality in this population [6,7], HBR appears to be a criterion that defines an overall frail population; however, this is significantly under-investigated.

Currently, the paucity of data on this specific subset of patients, frequently excluded from large clinical trials, limits our comprehension of the role that HBR plays in LM PCI outcomes [8]. Recently, some data have been published by Chiarito et al. [9]; however, it is limited to a single-centre experience, and comparison with results from a large, multicentre experience would significantly strengthen the reported outcomes. Moreover, Kang et al. [10] have recently shown that HBR patients from a Korean national registry are at increased risk of a composite of ischaemic events. However, the study included all PCI patients rather than focusing specifically on LM disease, and differences in revascularization strategies—such as the lower use of CABG among Asian patients—may limit comparability across populations [11].

The present study aims to investigate HBR, defined by the Academic Research Consortium (ARC) HBR criteria [8], within a multicentre cohort of LM disease patients undergoing PCI and to assess the impact of HBR status on the clinical outcomes of LM PCI.

## 2. Materials and Methods

### 2.1. Study Population

**Figure 1:**
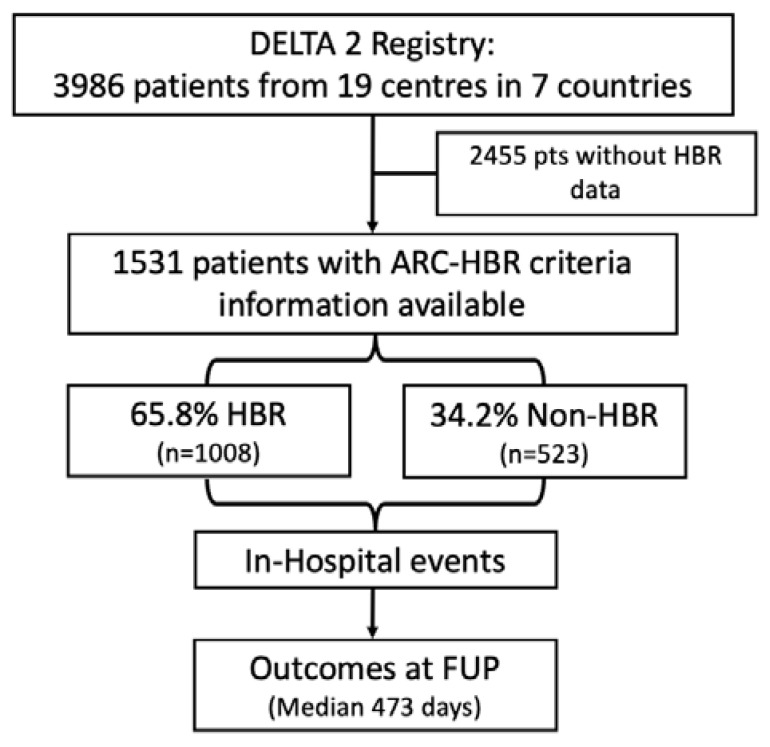
Study flowchart. HBR: high bleeding risk; ARC: academic research consortium; FUP: follow-up.

The present study is a subanalysis of the DELTA 2 Registry; the aims and outcomes are extensively described elsewhere [12]. Briefly, the DELTA 2 Registry included 3986 patients from 19 centres in 6 countries and aimed to investigate the outcomes of patients who underwent unprotected LM PCI with new-generation drug-eluting stents (DESs). From the 3986 patients in the DELTA2 registry, those for whom information on the bleeding risk status was lacking were excluded from the present analysis. Figure 1 represents the study flowchart for this subanalysis. The study complied with the Declaration of Helsinki and was approved by local ethics committees. All patients signed an informed written consent for participation in the registry.

### 2.2. Definitions and Endpoints

The Academic Research Consortium’s high bleeding risk definition was used to classify the patients’ bleeding risk profile, and patients were defined as high bleeding risk if presenting with at least one major criterion or at least two minor criteria [8]. Conversely, patients meeting one minor criterion, or no criteria, were defined as low bleeding risk. The adaptation of ARC HBR criteria to meet data availability was performed in line with the published literature [9] to ease the comparison of outcomes, and details are available in Supplementary Table S1.

Supplementary Table S2 provides a comparison between the baseline clinical characteristics of the patients included in the study and those belonging to the Delta 2 Registry who were excluded due to a lack of data on the HBR status.

The primary study objective was a composite of all-cause mortality, myocardial infarction (MI), and cerebrovascular accidents (CVA—as per DELTA 1 definition [3]) at follow-up. Secondary outcomes included individual components of the primary outcome, major and minor bleeding according to the TIMI (thrombolysis in myocardial infarction) classification [13], target vessel revascularization (TVR), target lesion revascularization (TLR), and probable or definite stent thrombosis. The study definitions are consistent with those of the previously published Delta Registry [3]; probable or definite stent thrombosis is defined according to the ARC criteria [14].

### 2.3. Statistical Analysis

Continuous variables are reported as mean ± standard deviation or median (interquartile range) and were compared using Student’s t-test or the Mann–Whitney U or Wilcoxon test, as appropriate. Categorical variables are reported as numbers (percentages) and were compared using the chi-square test or the Fisher exact test, as appropriate. The cumulative incidence of primary and secondary endpoints at follow-up was assessed using the Kaplan–Meier method and compared between groups using the log-rank test for time to the first event. Estimated risks are expressed as unadjusted and adjusted HRs and 95% CIs. Adjusted HRs were calculated using multivariable Cox regression, including the model the following variables: sex, congestive heart failure (CHF), and clinical presentation with acute coronary syndrome (ACS). Included variables were statistically significant in univariate analysis, and collinear variables (i.e., minor or major criteria for high bleeding risk) were not considered when formulating the regression model. A two-sided *p*-value < 0.05 defined statistical significance. The analysis was conducted using SPSS version 29.0 (IBM, Armonk, NY, USA).

## 3. Results

A total of 1531 patients were included in the analysis (38.4% of the overall Delta 2 study population); the mean age was 71.1 ± 10.8, and 72.8% were male. Among these, 1008 (65.8%) were at HBR according to the ARC definition, whereas 523 (34.2%) were not. Baseline characteristics and clinical presentation are described in Table 1. HBR patients were older, more frequently female, and affected by a higher burden of comorbidities, such as moderate, severe, and dialysis-dependent chronic kidney disease, insulin-dependent diabetes mellitus, moderate or severe anemia, peripheral artery disease, cerebrovascular disease, and chronic obstructive pulmonary disease. HBR patients presented more frequently with acute coronary syndrome (ACS): 22.6% of HBR patients presented with non-ST-segment elevation myocardial infarction (NSTEMI), and 9.2% presented with ST-segment elevation myocardial infarction (STEMI), compared to 4.8% and 3.1%, respectively, in the non-HBR population. HBR patients also had a lower left ventricular ejection fraction (LVEF) at baseline (HBR patients: 50.02 ± 13.0 vs. non-HBR: 55.6 ± 10.6, *p* < 0.001).

Procedural characteristics are reported in Supplementary Table S3. HBR patients had a significantly higher mean Syntax Score (28.9 ± 10.8 vs. 26.6 ± 10.9, *p* = 0.002), were more frequently treated in an urgent or emergent setting (305 [30.3%] vs. 31 [5.9%], *p* < 0.001), and received more intraprocedural mechanical circulatory support with an intra-aortic balloon pump (IABP) (108 [10.7%] vs. 16 [3.1%], *p* < 0.001).

**Figure 2:**
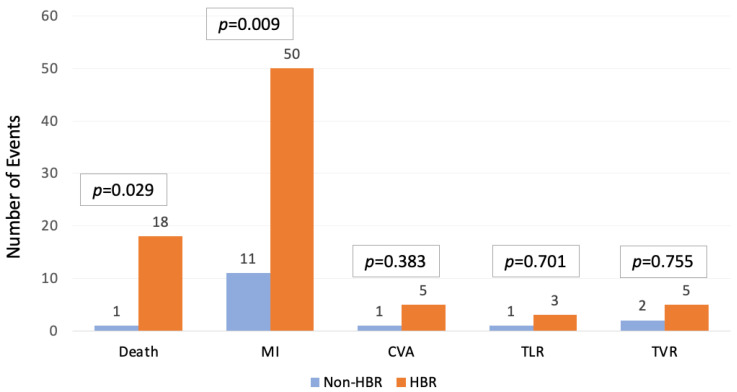
In-hospital outcomes. HBR patients showed a significantly higher incidence of in-hospital death and myocardial infarction (MI). MI: myocardial infarction, CVA: cerebrovascular accident, TLR: target lesion revascularization, TVR: target vessel revascularization, HBR: high bleeding risk.

In-hospital outcomes are reported in Table 2 and Figure 2. HBR patients experienced higher risk of in-hospital major complications, such as all-cause death (18 [1.8%] vs. 1 [0.2%], HR [CI]:9.5 [1.3–71.3]), myocardial infarction (50 [5.0%] vs. 11 [2.1%], HR [CI]:2.4 [1.3–4.7]), and acute kidney injury (15 [1.5%] vs. 2 [0.4%], HR [CI]:6.6 [1.5–29.0]. Procedure-specific outcomes such as TVR, TLR, and definite or probable stent thrombosis were comparable between the two groups.

Table 3 describes the medications prescribed at discharge. HBR patients were less frequently discharged on potent antiplatelet therapies (i.e., DAPT combinations with Ticagrelor and Prasugrel), and the difference was mainly driven by a lower prescription rate of Prasugrel (92 [9.3%] vs. 74 [14.2%], *p* = 0.004).

**Figure 3:**
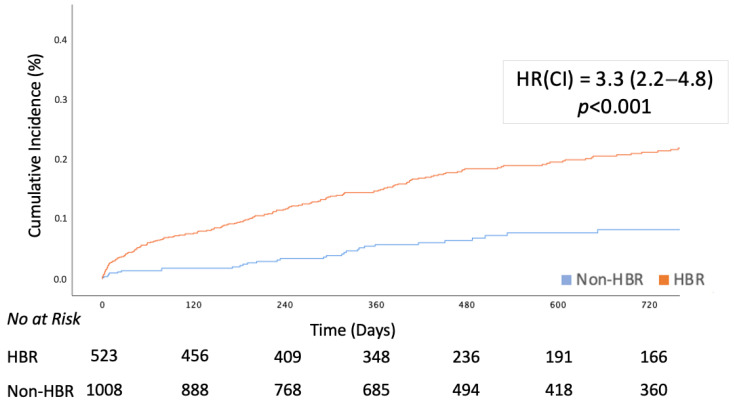
Cumulative incidence of the primary endpoint (all-cause death, myocardial infarction, and stroke). HBR patients had a significantly higher cumulative incidence of the composite endpoint (*p* < 0.001). HBR: high bleeding risk.

The outcomes at follow-up are described in Table 4 and Figure 3. The median follow-up was 473 (IQR: 301–931) days, and follow-up data were available for all patients. The composite primary endpoint occurred in 210 (20.8%) HBR patients and 32 (6.1%) non-HBR patients (HR [CI]:2.4 [1.6–3.4]) and was mainly driven by all-cause death (16.2% vs. 4.6%, HR [CI]:3.3 [2.2–5.1]) and myocardial infarction (5.8% vs. 1.9%, HR [CI]:2.9 [1.5–5.7]). Most deaths were ascribable to cardiovascular causes. TVR, TLR, and definite or probable stent thrombosis were comparable between the two groups. The incidence of the composite primary objective and all-cause death remained significantly higher in the multiple regression model, adjusted for sex, congestive heart failure, and acute coronary syndrome at presentation, whereas myocardial infarction did not.

## 4. Discussion

To the best of our knowledge, this work represents the largest multicentre study to investigate the prevalence and clinical outcomes of HBR patients undergoing LM PCI.

The main findings of the present study are as follows:

1. The prevalence of HBR is high among patients undergoing LM PCI.
2. HBR patients experienced worse in-hospital clinical outcomes (all-cause mortality, myocardial infarction, and acute kidney injury), whereas the incidence of procedural outcomes (TLR, TVR, definite or probable stent thrombosis) was low and comparable between the two groups.
3. The composite primary study objective occurred three times more frequently in the HBR population and was mainly driven by all-cause death according to adjusted Cox regression analysis; the procedural outcomes were durable and comparable between the two populations.

Historically, randomized clinical trials on LM revascularization were limited to patients at low surgical risk. Thus, older and frail patients with multiple comorbidities, who have an increased prevalence of high bleeding risk, were excluded [3,15,16]. In the SYNergy study between percutaneous coronary intervention with TAXus and cardiac surgery (Syntax), previous CABG or PCI and acute MI were considered specific exclusion criteria, along with discretional criteria such as age, low ejection fraction, and need for mechanical support with an intra-aortic balloon pump [17]. Similarly, previous PCI and CABG, inability to undergo DAPT, and life expectancy <3 years were exclusion criteria for the Everolimus-Eluting Stents or Bypass Surgery for Left Main Coronary Artery Disease (EXCEL) Trial [18]. As a result, there is currently limited data available on the prevalence and outcomes of HBR patients undergoing LM PCI, and the available data might not be representative of this numerous subgroup of patients. However, considering both patient selection, which tends to direct fitter patients to CABG, and epidemiological factors such as the general ageing of the population, the subgroup of elderly or fragile patients referred to PCI for left main disease is increasing. This trend is confirmed by a previously performed analysis on the DELTA-2 Registry, which also highlighted their increased ischaemic and bleeding risk [12]. The high prevalence of frail and comorbid patients among those who are directed towards PCI for LM disease underscores the need for more data regarding outcomes and complications to achieve the best balance between potential risks and benefits of treatment.

Our results suggest that HBR, as defined by the ARC criteria, may represent not only a bleeding risk but also a broader marker of frailty and vulnerability, as indicated by the higher rates of all-cause mortality and myocardial infarction in this group. The primary outcome was largely driven by all-cause death, implying that adverse events may extend beyond bleeding. Nonetheless, the lack of systematic bleeding data during follow-up limits the ability to directly assess the relationship between ARC-HBR status and actual bleeding events. Although this correlation is widely explored in the literature, this limitation should be considered when interpreting the prognostic value of HBR in this specific group of patients.

Despite the higher clinical risk profile of HBR patients, our study highlights that peri-procedural outcomes related to the PCI itself, such as TLR, TVR, and stent thrombosis, were not significantly different between HBR and non-HBR patients. This suggests that LM PCI remains a safe and effective treatment option for HBR patients, even in the presence of complex clinical and anatomical characteristics. The comparable procedural outcomes also indicate that contemporary PCI techniques, when performed in experienced centres, provide durable results irrespective of HBR status.

In this intricate scenario, the selection of revascularization strategies and antiplatelet therapies necessitates customization based on individual patient characteristics, and efforts to collect additional data on HBR patients undergoing PCI are warranted. The safety and feasibility of short-duration DAPT in HBR patients treated with next-generation drug-eluting stents have been demonstrated, but the applicability of these findings to LM PCI remains uncertain due to the underrepresentation of LM disease in these studies. Furthermore, recent observational and randomized studies have questioned the prognostic significance of thrombotic events complicating lesions situated in the LM [19,20]. Observational data, such as those from the IDEAL-LM trial, provide valuable insights but are limited by small sample sizes and methodological heterogeneity [21].

Our findings are consistent with recent evidence suggesting that the worse outcomes observed in HBR patients are largely attributable to their frailty and comorbidities rather than the procedural aspects of PCI [9]. These results underscore the need for future research to focus on strategies aimed at improving the overall prognosis of this high-risk population, including optimization of medical therapies and peri-procedural care.

The diagnosis of HBR should be regarded as a marker of overall frailty in high-risk patients. However, with appropriate care, PCI can offer a safe and effective treatment option as part of a comprehensive and balanced management strategy.

### Study Limitations

Our study has certain limitations that warrant acknowledgment. Firstly, the retrospective nature of the study rendered it impractical to delineate all 20 criteria for HBR, and the adaptation of some ARC HBR definition parameters was required to allow for alignment with the data available in our dataset. Moreover, the unavailability of some criteria might have led to an underestimation of the actual prevalence of HBR. Additionally, 2455 patients were excluded from the analysis due to missing data necessary for HBR classification. These excluded patients were generally younger and had fewer comorbidities and different clinical presentations, potentially introducing selection bias, which may affect the generalizability of our findings. As this study constitutes a subanalysis of a larger prospective registry with prespecified endpoints, data regarding bleeding outcomes and DAPT discontinuation were not complete enough to be reported in the analysis; however, the lack of this information does not hamper the conclusions that this work aims to deliver. The intrinsic overlap between HBR-defining criteria and baseline characteristics precluded the use of propensity score matching; therefore, the limited control for confounding factors should be considered when interpreting the independent prognostic impact of HBR. Finally, data from the DELTA 2 Registry refers to patients treated from 2006 to 2015 and followed for a median of 17 months. While treatment strategies and clinical pathways may have evolved since then, the widespread use of DESs and the availability of all modern antiplatelet options ensure that the findings of our study remain relevant and applicable to contemporary practice.

## 5. Conclusions

Individuals at high bleeding risk represent a high percentage of patients undergoing LM PCI. The worse outcomes at follow-up are mainly driven by all-cause death, whereas procedure-related outcomes, such as repeated revascularization and stent thrombosis, are comparable to those of the general population.

## Figures and Tables

**Table 1:** Baseline characteristics and clinical presentation.

	Overall n = 1531	HBR n = 1008 (65.8)	Non-HBR n = 523 (34.2)	*p*-Value
**Baseline Characteristics**				
Age	71.1 ± 10.8	73.7 ±10.8	66.1 ± 9.1	**<0.001**
Age > 75	626 (40.9)	555 (55.1)	71 (13.6)	**<0.001**
Male	1114 (72.8)	680 (67.5)	434 (83.0)	**<0.001**
BMI kg/m^2^	26.4 ± 5.3	26.1 ± 5.0	26.9 ± 5.8	0.338
Current smoker	230 (15.0)	126 (12.5)	104 (19.9)	**<0.001**
Hypertension	1251 (81.7)	804 (79.8)	447 (85.5)	**0.006**
Diabetes mellitus	588 (38.4)	380 (37.7)	208 (39.8)	0.429
Insulin dependent	163 (10.6)	125 (12.4)	38 (7.3)	**0.002**
Chronic kidney disease	614 (40.6)	549 (55.4)	65 (12.4)	**<0.001**
Severe CKD	118 (8.1)	118 (12.6)	-	**<0.001**
Hemodialysis	61 (5.2)	60 (9.2)	1 (0.2)	**<0.001**
Moderate anemia	211 (15.7)	143 (17.4)	68 (13.0)	**0.032**
Severe anemia	536 (39.8)	536 (65.0)	-	**<0.001**
CHF at presentation	102 (15.0)	94 (17.5)	8 (5.6)	**<0.001**
Previous MI	468 (30.6)	333 (33.1)	135 (25.8)	**0.003**
Previous PCI	578 (37.8)	363 (36.0)	215 (41.1)	0.053
Previous CABG	88 (5.8)	68 (6.8)	20 (3.8)	**0.020**
Family history of CAD	352 (23.9)	222 (23.3)	130 (24.9)	0.508
Peripheral arterial disease	298 (19.7)	238 (24.1)	60 (11.5)	**<0.001**
Cerebrovascular disease	214 (14.0)	198 (19.7)	16 (3.1)	**<0.001**
COPD	212 (14.7)	183 (19.0)	29 (6.0)	**<0.001**
**Clinical Presentation**				
Stable/silent ischemia	895 (58.5)	512 (50.8)	383 (73.2)	**<0.001**
Unstable angina	274 (17.9)	175 (17.4)	99 (18.9)	0.448
NSTEMI	253 (16.5)	228 (22.6)	25 (4.8)	**<0.001**
STEMI	109 (7.1)	93 (9.2)	16 (3.1)	**<0.001**
LVEF %	52.6 ± 12.3	50.2 ± 13.0	55.6 ± 10.6	**<0.001**

BMI: body mass index, CKD: chronic kidney disease, MI: myocardial infarction, PCI: percutaneous coronary intervention, CABG: coronary artery bypass grafting, CAD: coronary artery disease, COPD: chronic obstructive pulmonary disease, NSTEMI: non-ST-elevation myocardial infarction, STEMI: ST-elevation myocardial infarction, LVEF: left ventricular ejection fraction.

**Table 2:** In-hospital outcomes.

	Overall n = 1531	HBR n = 1008 (65.8)	Non-HBR n = 523 (34.2)	HR (95% CI)	*p*-Value
In-hospital death	19 (1.2)	18(1.8)	1 (0.2)	9.5 (1.3–71.3)	**0.029**
In-hospital MI	61 (4.0)	50 (5.0)	11 (2.1)	2.4 (1.3–4.7)	**0.009**
In-hospital CVA events	6 (0.4)	5 (0.5)	1 (0.2)	2.2 (0.3–22.3)	0.383
In-hospital TLR	4 (0.3)	3 (0.3)	1 (0.2)	1.6 (0.2–15.0)	0.701
In-hospital TVR	7 (0.5)	5 (0.5)	2 (0.4)	1.3 (0.3–6.7)	0.755
In-hospital MACCE	76 (5.0)	63 (6.3)	13 (2.5)	2.6 (1.4–4.8)	**0.002**
Definite/probable ST	6 (0.4)	5 (0.5)	1 (0.2)	2.2 (0.3–22.3)	0.383
In-hospital Infectious Complications	1 (1.0)	6 (1.6)	1 (0.3)	5.4 (0.7–45.2)	0.119
In-hospital AKI	17 (1.1)	15 (1.5)	2 (0.4)	6.6 (1.5–29)	**0.013**

MI: myocardial infarction, CVA: cerebrovascular accident, TLR: target lesion revascularization, TVR: target vessel revascularization, MACCE: major cardiovascular and cerebrovascular events, ST: stent thrombosis, AKI: acute kidney injury.

**Table 3:** Medications at discharge.

A	Overall n = 1531	HBR n = 1008 (65.8)	Non-HBR n = 523 (34.2)	*p*-Value
**Medications at discharge**				
Aspirin	1513 (98.8)	992 (98.4)	521 (99.6)	0.038
Clopidogrel	1263 (82.5)	860 (85.3)	403 (77.1)	**<0.001**
Ticagrelor	82 (5.4)	48 (4.9)	34 (6.5)	0.180
Prasugrel	166 (11.0)	92 (9.3)	74 (14.2)	**0.004**
OAC	92 (6.5)	92 (10.3)	-	**<0.001**
DAPT only	1403 (91.6)	890 (88.3)	513 (98.9)	**<0.001**
SAPT only	17 (1.5)	8 (1.3)	9 (1.7)	0.493
DAPT + OAC	83 (7.7)	83 (8.2)	-	**<0.001**
SAPT + OAC	9 (0.6)	9 (0.9)	-	**0.030**

OAC: oral anticoagulant, DAPT: double antiplatelet therapy, SAPT: single antiplatelet therapy.

**Table 4:** Cox regression analysis for outcomes at follow-up.

	Overall n = 1531	HBR n = 1008 (65.8)	Non-HBR n = 523 (34.2)	HR (95% CI)	*p*-Value	Adjusted HR (95% CI)	*p*-Value
Death or MI or CVA	242 (15.8)	210 (20.8)	32 (6.1)	3.3 (2.2–4.8)	<0.001	2.4 (1.6–3.4)	**<0.001**
All-Cause Death	187 (12.2)	163 (16.2)	24 (4.6)	3.3 (2.2–5.1)	<0.001	2.4 (1.5–3.8)	**<0.001**
Cardiovascular Death	142 (9.3)	124 (12.3)	18 (3.4)	3.2 (1.95–5.26)	<0.001	3.5 (1.1–11.1)	**0.038**
MI	68 (4.4)	58 (5.8)	10 (1.9)	2.9 (1.5–5.7)	0.002	2.1 (0.9–4.4)	0.062
CVA	19 (1.2)	17 (1.7)	2 (0.4)	4.2 (0.9–18.3)	0.054	3.3 (1.2–23.6)	0.029
Definite/Probable ST	30 (2.0)	23 (2.3)	7 (1.3)	1.6 (0.7–4.0)	0.22	1.0 (0.3–2.3)	0.737
TLR	162 (10.6)	104 (10.3)	58 (11.1)	0.9 (0.7–1.3)	0.572	1.0 (0.7–1.5)	0.805
TVR	265 (17.3)	152 (15.1)	113 (21.6)	0.6 (0.5–0.8)	<0.001	0.8 (0.6–1.0)	0.095

CVA: cerebrovascular accident, MI: myocardial infarction, ST: stent thrombosis, TLR: target lesion revascularization, TVR: target vessel revascularization.

## Data Availability

The data supporting the results presented in this research are available upon reasonable request.

## Supplementary Materials

The following supporting information can be downloaded at https://www.mdpi.com/article/10.3390/jcdd12050179/s1, Supplementary Table S1: Adaptation of ARC-HBR criteria and prevalence within the study population, Supplementary Table S2: Comparison between baseline clinical characteristics of Delta 2 patients who were included and excluded from the study, Supplementary Table S3: Procedural characteristics.
