# Supplementary material for: Percutaneous Coronary Intervention for Left Main Disease in High Bleeding Risk: Outcomes from a Subanalysis of the Delta 2 Registry
Source: J Cardiovasc Dev Dis. 2025 May 11;12(5):179. doi: 10.3390/jcdd12050179 (PMC12112215; doi:10.3390/jcdd12050179)
Supplement: Supplementary file 1 [file jcdd-12-00179-s001.zip › jcdd-3439768-supplementary.pdf]

Supplementary material

Supplementary Table S1: adaptation of ARC-HBR criteria and prevalence within the study population

ARC-HBR criteria	Study definition	ARC-HBR document definition[1]	HBR n=1008 (65.8)	Non-HBR n=523 (34.2)
**Major Criteria**				
Oral anticoagulation	Oral anticoagulation at discharge of hospitalization for PCI	Anticipated use of long-term oral anticoagulation	92 (10.3)	-
Severe/end-stage CKD	eGFR <30 mL/min	No difference	118 (12.6)	-
Moderate/severe anaemia	Haemoglobin <11 g/dL	No difference	536 (65.0)	-
Thrombocytopenia	NA	Platelet count <100 x10^9/L		
Malignancy	NA	Active malignancy within the past 12 months		
Planned surgery	NA	Non-deferrable major surgery on dual antiplatelet therapy		
	NA	Spontaneous bleeding in the past 6 months or at any time, if recurrent		
	NA	Chronic bleeding diathesis		
	NA	Liver cirrhosis with portal hypertension		
	NA	Previous spontaneous ICH; traumatic ICH within the past 12 months; presence of bAVM; ischemic stroke within the past 6 months		
	NA	Recent major surgery or major trauma within 30 days before PCI		
**Minor criteria**				
Age 75+	Age ≥75 years	No difference	555 (55.1)	71 (13.6)
Moderate CKD	eGFR 30–59 mL/min	No difference	429 (45.8)	66 (12.6)
Mild anemia	Hemoglobin 11–12.9 g/dL for men and 11–11.9 g/dL for women	No difference	143 (17.4)	68 (13.0)
Prior bleeding	NA	Spontaneous bleeding within the past 12 months not meeting the major criterion		
Prior CVA	History of cerebrovascular disease	Any ischemic stroke at any time not meeting the major criterion	198 (19.7)	16 (3.1)
	NA	Long-term use of oral NSAIDs or steroids		

ARC-HBR = Academic Research Consortium for High Bleeding Risk; bAVM = brain arteriovenous malformation; CKD = chronic kidney disease; CVA = cerebrovascular accident; eGFR = estimated glomerular filtration rate; ICH = intracranial hemorrhage; NA = not available; NSAIDs = nonsteroidal anti-inflammatory drugs; PCI = percutaneous coronary intervention

[1] Definitions adapted from Urban P. et al., Circulation 2019;140:240-261.

Supplementary Table S2: Comparison between baseline clinical characteristics of Delta 2 patients who were included and excluded from the study

	Pts included in the analysis n=1531	Pts excluded from the analysis n=2455	p-Value
**Baseline Characteristics**			
Age	71.1 ± 10.8	68.6 ± 10.8	0.020
Male	1114 (72.8)	1855 (75.6)	<0.049
BMI Kg/m^2	26.4 ± 5.3	26.6 ± 4.5	0.338
Current smoker	230 (15.0)	400 (16.3)	0,285
Hypertension	1251 (81.7)	1867 (76.0)	<0.001
Diabetes mellitus	588 (38.4)	638 (26.0)	<0.001
Chronic kidney disease	614 (40.6)	585 (23.8)	<0.001
Haemodialysis	61 (5.2)	32 (1.3)	<0.001
Previous MI	468 (30.6)	647 (26.5)	0.009
Previous PCI	578 (37.8)	1061 (43.4)	<0.001
Previous CABG	88 (5.8)	241 (9.8)	<0.001
Family history of CAD	352 (23.9)	707 (31.9)	<0.001
Multivessel disease	1109 (72.4)	1853 (75.5)	0.033
Peripheral arterial disease	298 (19.7)	313 (12.7)	0.003
Cerebrovascular disease	214 (14.0)	158 (6.4)	<0.001
**Clinical Presentation**			
Stable/silent ischaemia	895 (58.5)	1651 (67.3)	<0.001
Unstable angina	274 (17.9)	330 (13.4)	<0.001
NSTEMI	253 (16.5)	335 (13.6)	<0.013
STEMI	109 (7.1)	139 (5.7)	<0.064
LVEF %	52.6 ± 12.3	53.9 ± 10.4	<0.001

BMI: body mass index, CKD: chronic kidney disease, MI: myocardial infarction, PCI: percutaneous coronary intervention, CABG: coronary artery bypass grafting, CAD: coronary artery disease, COPD: chronic obstructive pulmonary disease, NSTEMI: non st-elevation myocardial infarction, STEMI: ST-elevation myocardial infarction, LVEF: left ventricular ejection fraction

Supplementary Table S3: Procedural Characteristics

	Overall n=1531	HBR n=1008 (65.8)	Non-HBR n=523 (34.2)	p-Value
**Disease status**				
Ostial LM stenosis	261 (17.0)	183 (18.1)	78 (14.9)	0.110
Distal LM stenosis	784 (51.2)	492 (48.8)	292 (55.8)	0.009
Stenosis of ostial and distal LM	486 (31.7)	333 (33.0)	153 (29.3)	0.132
True Bifurcation	612 (48.2)	387 (46.9)	225 (50.6)	0.214
LAD/LCX disease	1311 (85.6)	877 (87.0)	434 (83.0)	0.033
RCA disease	689 (45.0)	494 (49.0)	195 (37.3)	<0.001
In-stent restenosis	66 (4.5)	37 (3.9)	29 (5.6)	0.140
Euroscore II				
Syntax Score		28.9±10.8	26.6 ±10.9	0.002
**PCI details**				
Urgent or emergent setting	336 (21.9)	305 (30.3)	31 (5.9)	<0.001
Radial Approach	257 (16.8)	191 (18.9)	66 (12.6)	0.002
PCI of ostial LM	278 (18.2)	188 (18.7)	90 (17.2)	0.487
PCI of distal LM/bifurcation	1253 (81.8)	820 (81.3)	433 (82.8)	0.487
Use of IVUS	684 (44.7)	441 (43.8)	243 (46.5)	0.311
N Vessels treated				<0.001
1	594 (40.3)	409 (43.0)	185 (35.4)	
2	578 (39.2)	380 (39.9)	198 (37.9)	
3	295 (20.0)	156 (16.4)	139 (26.6)	
4	7 (0.5)	6 (0.6)	1 (0.2)	
5	1 (0.1)	1 (0.1)	-	
Rotablation	171 (11.4)	102 (10.4)	69 (13.3)	0.089
Predilatation	667 (56.4)	410 (61.9)	257 (49.4)	<0.001
**Stent technique**				0.049
One stent/provisional single	1004 (77.3)	689 (77.5)	315 (76.8)	
Any crush	87	48	39	
T-stenting, TAP-two stent provisional	131(10.1)	96 (10.8)	35 (8.5)	
Culotte	64 (4.9)	46 (5.2)	18 (4.4)	
V-stenting	13 (1.0)	10 (1.1)	3 (0.7)	
Any 2 stent technique	295 (22.7)	200 (22.5)	95 (23.2)	
Kissing balloon inflation	575 (44.3)	379 (42.6)	196 (47.8)	0.081
Post-dilatation	1246 (81.4)	804 (79.8)	442 (84.5)	0.024
IABP use	124 (8.1)	108 (10.7)	16 (3.1)	<0.001

LM: left main, LAD: left anterior descending artery, LCX: left circumflex artery, RCA: right coronary artery, PCI: percutaneous coronary intervention, IVUS: intravascular ultrasound, TAP: t-and-protrusion, IABP: intra-aortic balloon pump
